# Futile complete recanalization: patients characteristics and its time course

**DOI:** 10.1038/s41598-020-61748-y

**Published:** 2020-03-18

**Authors:** Takaya Kitano, Kenichi Todo, Shinichi Yoshimura, Kazutaka Uchida, Hiroshi Yamagami, Nobuyuki Sakai, Manabu Sakaguchi, Hajime Nakamura, Haruhiko Kishima, Hideki Mochizuki, Masayuki Ezura, Yasushi Okada, Kazuo Kitagawa, Kazumi Kimura, Makoto Sasaki, Norio Tanahashi, Kazunori Toyoda, Eisuke Furui, Yuji Matsumaru, Kazuo Minematsu, Takeshi Morimoto

**Affiliations:** 1 Stroke Center, Osaka University Hospital, Osaka, Japan; 2 Department of Neurosurgery, Hyogo College of Medicine, Hyogo, Japan; 3 Division of Stroke Care Unit, National Cerebral and Cardiovascular Center, Osaka, Japan; 4 Department of Neurosurgery, Kobe City Medical Center General Hospital, Hyogo, Japan; 5 Department of Neurosurgery, National Hospital Organization Sendai Medical Center, Miyagi, Japan; 6 Department of Cerebrovascular Medicine and Neurology, National Hospital Organization Kyushu Medical Center, Fukuoka, Japan; 7 Department of Neurology, Tokyo Women’s Medical University, Tokyo, Japan; 8 Department of Neurology, Graduate School of Medicine, Nippon Medical School, Tokyo, Japan; 9 Institute for Biomedical Sciences, Iwate Medical University, Iwate, Japan; 10 Department of Neurology, Saitama Medical University International Medical Center, Saitama, Japan; 11 Department of Cerebrovascular Medicine, National Cerebral and Cardiovascular Center, Osaka, Japan; 12 Department of Stroke Neurology, Saiseikai Toyama Hospital, Toyama, Japan; 13 Division of stroke prevention and treatment, Department of Neurosurgery, Faculty of Medicine, University of Tsukuba, Ibaraki, Japan; 14 Department of Clinical Epidemiology, Hyogo College of Medicine, Hyogo, Japan

**Keywords:** Stroke, Stroke, Cardiology

## Abstract

As the goal of mechanical thrombectomy is shifting toward mTICI-3 rather than mTICI-2b, we sought to clarify the limitation of the effect of mTICI-3. A post-hoc analysis of a registry of large-vessel occlusion stroke from 46 centers was conducted. Among 2,420 registered patients, 725 patients with anterior circulation occlusion who achieved successful reperfusion were analyzed. We compared outcomes between patients with mTICI-3 and mTICI-2b, and investigated how the effect of mTICI-3 changed according to baseline characteristics and time course. The proportion of patients with favorable outcomes (mRS 0–2 at day 90) was higher among patients with mTICI-3 compared to those with mTICI-2b (adjusted OR, 2.10; 95% CI, 1.49–2.97). There was no heterogeneity in the effect of mTICI-3 with respect to age, neurological deficit, alteplase use, occluded vessels, or infarct size. mTICI-3 was associated with favorable outcomes when the puncture-to-reperfusion time was <80 minutes (adjusted OR, 2.28; 95% CI, 1.52–3.41), but not when the puncture-to-reperfusion time was ≥80 minutes. A significant heterogeneity was found in the effect of mTICI-3 reperfusion across the puncture-to-reperfusion time subgroups (P for interaction = 0.025). Until when operators should continue the procedure after mTICI-2b has been achieved, needs to be studied.

## Introduction

Successful reperfusion in endovascular therapy (EVT) for acute ischemic stroke is commonly defined as modified thrombolysis in cerebral infarction (mTICI) scores of 2b or 3^1–3^. It has been shown that outcomes of patients with complete reperfusion (mTICI-3) are superior to those with incomplete reperfusion (mTICI-2b)^4–16^, and the importance of achieving mTICI-3 reperfusion is remarked. However, whether the superiority of mTICI-3 is retained in any patient subgroups has not been fully investigated. Particularly, how time course modifies the effect of mTICI-3 reperfusion needs to be clarified because time is a critical factor in EVT for acute ischemic stroke^17^.

In this study, we sought to identify the subgroup of patients in whom the effect of mTICI-3 reperfusion was absent. For this purpose, we examined how the effect of mTICI-3 reperfusion changes according to baseline characteristics and the time to reperfusion using a nationwide large data set of patients with acute large vessel occlusion stroke.

## Methods

### Ethic statement

This study complied with the Declaration of Helsinki guidelines for investigations involving humans, and all methods were carried out in accordance with relevant guidelines and regulations as for observational study. The study design and protocols were approved by the institutional review board of Osaka University Hospital. The need for written informed consent was waived, and the consent to participate in this study was obtained using an opt-out approach.

### Subjects

This study is a post-hoc analysis of the RESCUE-Japan Registry 2, which was a prospective, multicenter registry enrolling 2,420 patients with an acute cerebral large vessel occlusion in 46 centers in Japan between October 1, 2014 and September 30, 2016^17^. The institutional review boards of all participating centers approved the protocol (see Supplementary Methods for full name of institutional review boards of all participating centers). RESCUE-Japan Registry 2 was designed to clarify the generalizability of the effectiveness of EVT in real-world patients. During the study period, EVT for acute ischemic stroke was basically indicated for patients with large vessel occlusion stroke within 8 hours from a stroke. Patients with large infarction were basically excluded from the indication. The decision to perform EVT was left to the attending physician. In the present study, we analyzed patients with anterior circulation occlusion who underwent mechanical thrombectomy within 12 hours of onset.

### Imaging and endovascular therapy

Diagnostic modalities before EVT were not unified in the RESCUE-Japan Registry 2. The Alberta Stroke Program Early Computerized Tomography Score (ASPECTS) was derived from computed tomography (CT) or magnetic resonance imaging (MRI) data. If both CT and MRI were performed before EVT, the lower ASPECTS was used. The final mTICI scores were assessed by the attending interventionalists. A successful reperfusion was defined as a score of mTICI-2b or 3^2^. The puncture to reperfusion time was defined as the interval between time of groin puncture and time when reperfusion status reached the final state, and was recorded by 5 minute intervals.

### Outcomes

We set the modified Rankin Scale (mRS)^18^ of 0–1 and 0–2 at 90 days after stroke onset as excellent and favorable outcomes, respectively. The primary outcome of this study was the favorable outcome, and the secondary outcomes were the excellent outcome and mortality at 90 days after stroke onset. As safety outcomes, any intracranial hemorrhage and symptomatic intracranial hemorrhage within 72 h of EVT according to SITS-MOST criteria^19^ were considered.

### Statistical analysis

Patient characteristics and outcomes were compared between patients who achieved mTICI-3 and mTICI-2b reperfusion. These values are presented as median with interquartile range for continuous variables, and as numbers and percentages for the categorical variables. Continuous variables were compared using Wilcoxon rank sum test. Fisher’s exact test or chi-squared test was used for categorical variables, when appropriate. We constructed univariate and multivariate logistic regression models. Adjusted variables in the multivariate logistic regression model are as follows: age, sex, National Institutes of Health Stroke Scale (NIHSS) score, ASPECTS, target occlusion location (internal carotid artery, the horizontal segment of the middle cerebral artery, or other vessels), intravenous alteplase administration, and time from onset to reperfusion.

In order to assess the heterogeneity of the effect of complete reperfusion by baseline characteristics, subgroups were defined as follows: (1) age ≤ 75 or> 75 years^17^; (2) NIHSS score ≥10 or <10; (3) use or non-use of alteplase; (4) proximal (internal carotid artery or the horizontal segment of the middle cerebral artery) or distal (the insular segment or more peripheral segment of the middle cerebral artery, or anterior cerebral artery) vessel occlusion^20^; and (5) ASPECTS ≥ 6 or <6^20,21^. In each subgroup, we investigated the association between mTICI and clinical outcomes. The adjusted variables in multivariate logistic regression models were same as above.

To assess how the delay of reperfusion modifies the effect of complete reperfusion, we divided the onset-to-reperfusion time, the onset-to-puncture time, and the puncture-to-reperfusion time by their upper quartile. Then, we investigated the association between mTICI and clinical outcomes in each subgroup. The adjusted variables in multivariate logistic regression models were the same as above except these: puncture-to-reperfusion time was adjusted for in analyzing the subgroups of onset-to-puncture time; onset-to-puncture time was adjusted for in analyzing the subgroups of puncture-to-reperfusion time. Furthermore, we analyzed how the mTICI3 effect changes according to time to reperfusion in continuous valuables. We report graphically plots of predicted probabilities of functional outcomes stratified by mTICI.

The interaction was tested using a multiplicative interaction term (mTICI*variable) included in the models. The time course was used as both dichotomized and continuous variable. All reported P values were 2-tailed, and statistical significance was established at *P* < 0.05. We did all analyses using SAS University Edition (SAS Institute Inc, Cary, NC) and drew figures with JMP Pro 14.3 (SAS Institute Inc, Cary, NC).

## Results

### Population

**Figure 1:**
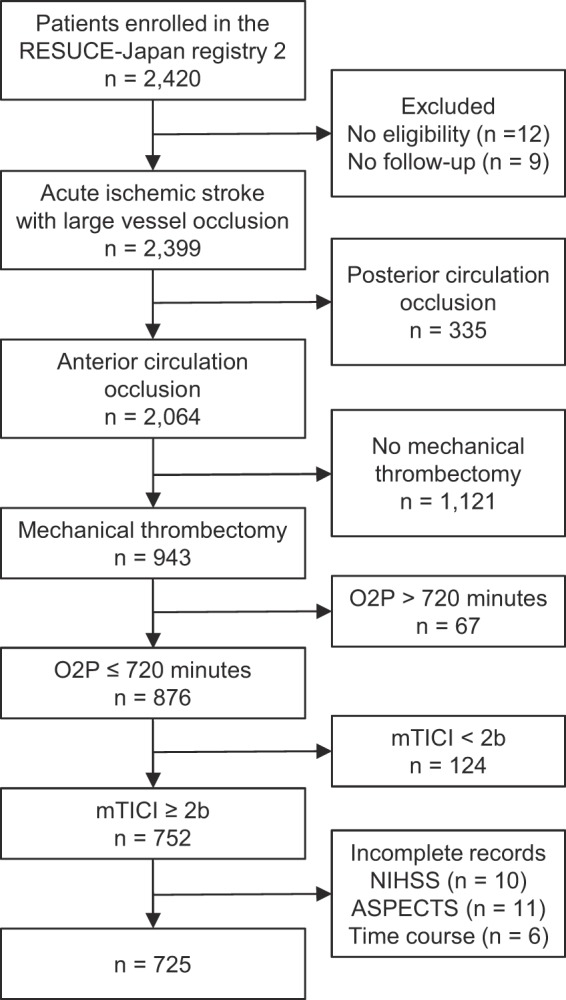
Patient selection. ASPECTS, Alberta Stroke Program Early Computerized Tomography Score; mTICI, modified thrombolysis in cerebral infarction; NIHSS, National Institutes of Health Stroke Scale; O2P, onset-to-puncture.

Among the 2,420 enrolled patients, 21 patients were excluded because 12 patients did not meet the eligibility criteria and 9 patients were lost to follow-up. Patients with posterior circulation occlusion were also excluded (n = 335). In the remaining 2,064 patients, 876 patients underwent EVT using stent retriever or aspiration catheter within 12 hours of onset. Among them, patients who achieved successful recanalization (mTICI-2b and 3) were included (n = 752). Of those patients, 27 were excluded due to incomplete records. As a result, a total of 725 patients were analyzed in the present study (Fig. 1).

### Baseline characteristics

Among the 725 patients analyzed, 389 patients (54%) achieved mTICI-3. Baseline characteristics are shown in Table 1. In patients with mTICI-3, median ASPECTS was higher than in those with mTICI-2b (8 versus 7, p = 0.006). Stent retrievers were used in 504 patients (70%), an aspiration catheter in 387 patients (53%), and both in 166 patients (23%). The upper quartiles of onset-to-reperfusion time, onset-to-puncture time, and puncture-to-reperfusion time were 360 minutes, 300 minutes, and 75 minutes, respectively. Onset-to-puncture time was similar, but puncture-to-reperfusion time was shorter in patients with mTICI-3 than those with mTICI-2b (40 versus 55 minutes, p < 0.001).

**Table 1:** Patient characteristics.

	Total (n = 725)	mTICI-3 (n = 389)	mTICI-2b (n = 336)	P-value
Age, years	76 (67–83)	75 (67–83)	76 (67–83)	0.68
Male gender	421 (58%)	223 (57%)	198 (59%)	0.66
Study period after March 2015	640 (88%)	342 (88%)	298 (89%)	0.82
Smoking	103 (14%)	55 (14%)	48 (14%)	1.0
Modified Rankin Scale score	0 (0–1)	0 (0–1)	0 (0–1)	0.95
Hypertension	413 (57%)	225 (58%)	188 (56%)	0.61
Diabetes	136 (19%)	74 (19%)	62 (19%)	0.85
Hyperlipidemia	162 (22%)	87 (22%)	75 (22%)	1.0
Atrial fibrillation	396 (55%)	214 (55%)	182 (54%)	0.82
NIHSS score	18 (14–23)	18 (14–22)	18 (14–23)	0.67
ASPECTS*	7 (6–9)	8 (6–9)	7 (6–9)	0.006
Site of occlusion				
ICA	257 (35%)	144 (37%)	113 (34%)	0.35
M1	347 (48%)	187 (48%)	160 (48%)	0.94
M2 or distal	130 (18%)	67 (17%)	63 (19%)	0.63
ACA	12 (1.7%)	2 (0.5%)	10 (3.0%)	0.016
Cardioembolic stroke	567 (78%)	309 (79%)	258 (77%)	0.42
Intravenous alteplase	374 (52%)	203 (52%)	171 (51%)	0.77
Use of stent retrievers	504 (70%)	267 (69%)	237 (71%)	0.63
Use of aspiration catheters†	387 (53%)	195 (50%)	192 (57%)	0.062
Onset-to-reperfusion, minutes	255 (185–360)	245 (175–350)	265 (195–384)	0.052
Onset-to-puncture, minutes	190 (130–300)	190 (125–295)	190 (135–313)	0.58
Puncture-to-reperfusion, minutes	45 (35–75)	40 (30–65)	55 (35–84)	<0.001

Data are presented as n (%) or median (interquartile range). ACA, anterior cerebral artery; ASPECTS, Alberta Stroke Program Early Computerized Tomography Score; ICA, internal carotid artery; mTICI, modified treatment in cerebral infarction; NIHSS, National Institutes of Health Stroke Scale; M1, the horizontal segment of the middle cerebral artery; and M2, the insular segment of the middle cerebral artery.

*If both CT and MRI were performed before treatment, the lower one was used.

^†^The utilization of an aspiration catheter as a distal access device was also counted.

### Overall analysis

A favorable outcome was observed significantly more often in patients with mTICI-3 than those with mTICI-2b (53% versus 37%; adjusted odds ratio [OR], 2.10; 95% confidence interval [CI], 1.49–2.97) (Table 2). An excellent outcome was also observed significantly more often in patients with mTICI-3 than those with mTICI-2b in univariate and multivariate analyses (36% versus 21%; adjusted OR, 2.23; 95% CI, 1.53–3.25). There was no significant difference in mortality between the two groups. The proportion of patients with any intracranial and symptomatic intracranial hemorrhages within 72 hours were lower in patients with mTICI-3 than those with mTICI-2b (24% versus 33%; adjusted OR, 0.71; 95% CI, 0.51–0.99, and 1.0% versus 3.6%; adjusted OR, 0.30; 95% CI, 0.09–0.94, respectively).

**Table 2:** Outcomes at 90 days.

Outcomes	mTICI-3 (n = 389)	mTICI-2b (n = 336)	Crude ORs (95% CI)	P	Adjusted ORs (95% CI)	P
Primary outcome						
mRS score 0–2	208 (53)	125 (37)	1.94 (1.44–2.61)	<0.001	2.10 (1.49–2.97)	<0.001
Secondary outcomes						
mRS score 0–1	139 (36)	69 (21)	2.15 (1.54–3.01)	<0.001	2.23 (1.53–3.25)	<0.001
Mortality	26 (6.7)	27 (8.0)	0.82 (0.47–1.43)	0.49	0.86 (0.49–1.52)	0.60
Safety outcomes						
Any ICH within 72 h	95 (24)	111 (33)	0.66 (0.47–0.91)	0.011	0.71 (0.51–0.99)	0.044
Symptomatic ICH within 72 h	4 (1.0)	12 (3.6)	0.28 (0.09–0.88)	<0.029	0.30 (0.09–0.94)	0.039

Data are presented as n (%). CI indicates confidence interval; ICH, intracranial hemorrhage; mRS, modified Rankin Scale; mTICI, modified thrombolysis in cerebral infarction; OR, odds ratio.

Adjusted variables are as follows: age, sex, National Institutes of Health Stroke Scale score, Alberta Stroke Program Early Computerized Tomography Score, target occlusion location, intravenous alteplase administration, and time from onset to reperfusion.

### Subgroup analyses based on baseline characteristics

**Figure 2:**
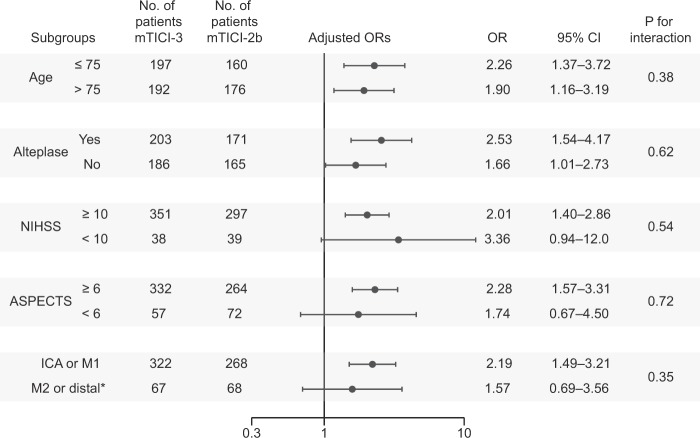
Adjusted odds ratios (ORs) of complete reperfusion (mTICI-3) for favorable outcome according to baseline characteristics. Adjusted for age, sex, NIHSS score, ASPECTS, target occlusion location (ICA, M1, or the other), intravenous alteplase administration, and onset-to-reperfusion time. *Five patients with anterior cerebral artery occlusion are included. NIHSS, National Institutes of Health Stroke Scale; ASPECTS, Alberta Stroke Program Early Computerized Tomography Score; CI, confidence interval; ICA, internal carotid artery; mTICI, modified thrombolysis in cerebral infarction; M1, the horizontal segment of the middle cerebral artery; M2, the insular segment of the middle cerebral artery.

The result of our subgroup analysis by baseline characteristics is summarized in a forest plot (Fig. 2). Favorable outcome was observed significantly more often in patients with mTICI-3 than in those with mTICI-2b in the subgroups of age ≤ 75, age > 75, alteplase use, no alteplase, NIHSS score ≥ 10, ASPECTS ≥ 6, and proximal vessel occlusion. There was no heterogeneity in the effect of mTICI-3 reperfusion on favorable outcomes with respect to age, use of alteplase, NIHSS score, ASPECTS, or target occlusion location.

The ORs for excellent outcome are shown in Supplementary Fig. S1. Excellent outcome was observed significantly more often in patients with mTICI-3 than in those with mTICI-2b in all the subgroups, except subgroups of NIHSS < 10 and ASPECTS < 6. There was no heterogeneity in the effect of mTICI-3 reperfusion on excellent outcomes with respect to the baseline characteristics.

### Subgroup analyses based on the time course

**Figure 3:**
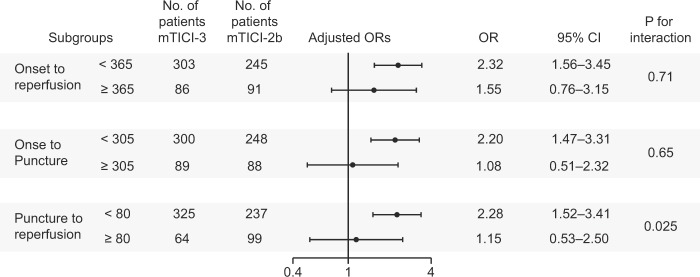
Adjusted odds ratios (ORs) of complete reperfusion (mTICI-3) for favorable outcome according to time course (onset-to-reperfusion, onset-to-puncture, and puncture-to-reperfusion time). These time variables are divided by the upper quartile. CI; confidence interval; mTICI, modified thrombolysis in cerebral infarction.

Baseline characteristics of each subgroup based on the time course are shown in Supplementary Tables S1, S2 and S3. The result of the subgroup analysis is summarized in a forest plot (Fig. 3). In the subgroup of onset-to-reperfusion time <365, onset-to-puncture time <305, puncture-to-reperfusion time <80, favorable outcome was observed significantly more often in patients with mTICI-3 than those with mTICI-2b (adjusted OR, 2.32; 95% CI, 1.56–3.45; adjusted OR, 2.20; 95% CI, 1.47–3.31; and adjusted OR, 2.28; 95% CI, 1.52–3.41, respectively). Contrarily, there was no significant association between mTICI score and favorable outcome in the subgroup of puncture-to-reperfusion time ≥80 (adjusted OR, 1.15; 95% CI, 0.53–2.50), and there was significant heterogeneity in the effect of mTICI-3 reperfusion across the puncture-to-reperfusion time subgroups (P for interaction = 0.025), suggesting that the effect of mTICI-3 reperfusion diminishes within the highest quartile of puncture-to-reperfusion time.

The ORs for excellent outcome are shown in Supplementary Fig. S2. In the subgroup of onset-to-reperfusion time <365, onset-to-puncture time <305, and puncture-to-reperfusion time <80, excellent outcome was observed significantly more often in patients with mTICI-3 than in those with mTICI-2b (adjusted OR, 2.35; 95% CI, 1.55–3.55; adjusted OR, 2.13; 95% CI, 1.39–3.26; and adjusted OR, 2.35; 95% CI, 1.53–3.61, respectively). On the other hand, there was no significant association between mTICI score and excellent outcome in the subgroup of puncture-to-reperfusion time ≥80 (adjusted OR, 1.17; 95% CI, 0.46–3.00), and there was a marginal heterogeneity in the effect of mTICI-3 reperfusion across the puncture-to-reperfusion time subgroups (P for interaction = 0.073).

**Figure 4:**
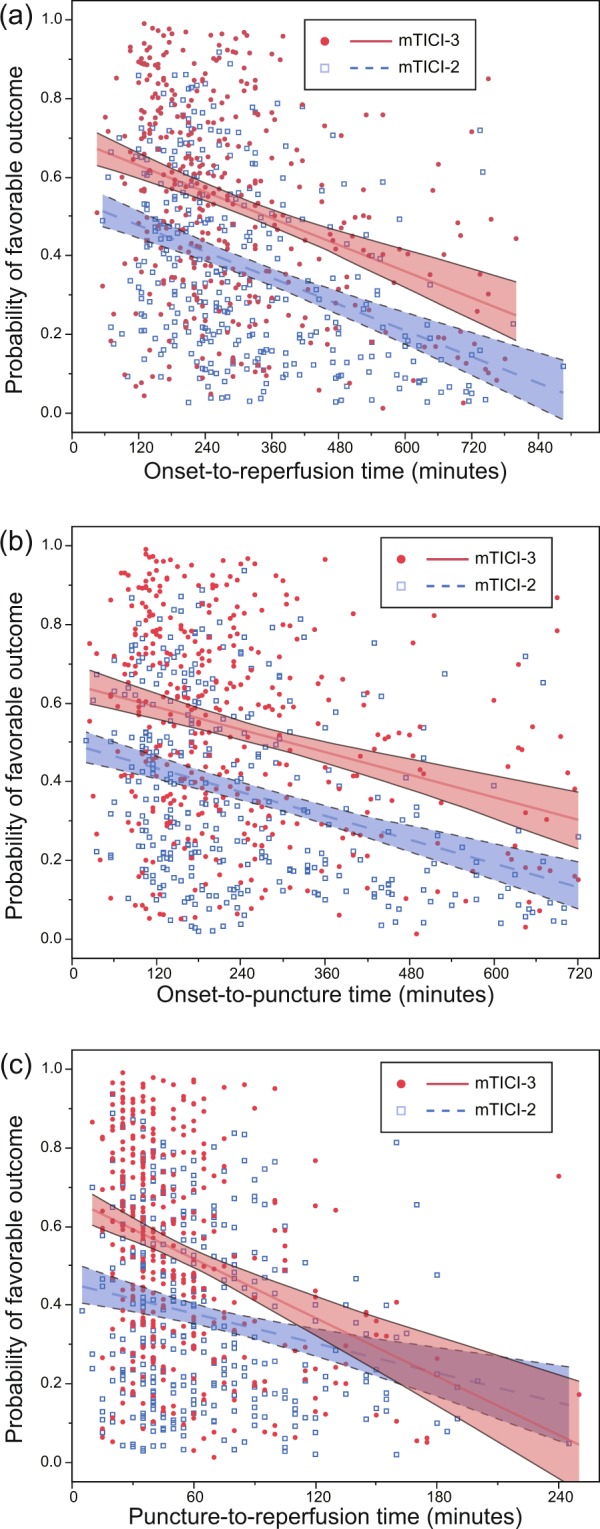
The probabilities of favorable outcome estimated using logistic regression with stratification by mTICI scores are plotted. The regression lines and 95% confidence intervals are shown. The time course was included as a continuous variable. (**a**) On onset-to-reperfusion time. Adjusted for baseline characteristics (age, sex, National Institutes of Health Stroke Scale score, Alberta Stroke Program Early Computerized Tomography Score, target occlusion location, intravenous alteplase). (**b**) On onset-to-puncture time. Adjusted for baseline characteristics and puncture-to-reperfusion time. (**c**) On puncture-to-reperfusion time. Adjusted for baseline characteristics and onset-to-puncture time.

Changes in the probability of favorable outcome with time course in continuous variables stratified by mTICI score are shown in Fig. 4. As a continuous variable, none of the onset-to-reperfusion time, onset-to-puncture time, or puncture-to-reperfusion time significantly modified the effect of complete reperfusion (P for interaction = 0.77, 0.70, 0.25, respectively). Changes in the probability of excellent outcome with time course in continuous variables stratified by mTICI score are shown in Supplementary Fig. S3. As a continuous variable, none of the time course variables significantly modified the effect of complete reperfusion.

## Discussion

Our study demonstrated that the effect of mTICI-3 reperfusion was not significantly modified by patients’ baseline characteristics such as age, sex, NIHSS score, ASPECTS, and target occlusion location among patients with anterior circulation occlusion. Previous studies have reported the effect of complete reperfusion on outcomes^4–12,22–24^, but the heterogeneity of this effect according to patients’ characteristics has not been fully investigated. Although the superiority of mTICI-3 reperfusion did not reach statistical significance in some subgroups, presumably due to insufficient power, our findings provide further support for the importance of achieving mTICI-3 reperfusion regardless of the baseline characteristics.

On the other hand, the association between mTICI-3 reperfusion and clinical outcome was not significant in patients who achieved mTICI-3 later than 80 minutes after the puncture, and the effect of complete reperfusion was modified by the puncture-to-reperfusion time ≥80 minutes. This is not surprising because the ischemic core grows into the penumbral region as time passes^25^, and the brain tissue that can be rescued becomes less. This study could not determine until when an operator should continue the procedure aiming for mTICI-3 reperfusion after mTICI-2b has been achieved; however, it is worth noting that long procedure time would limit the advantage of mTICI-3 reperfusion.

While a significant heterogeneity in the effect of mTICI-3 reperfusion was observed according to puncture-to-reperfusion time, whether the effect of mTICI-3 reperfusion changes by onset-to-puncture time remained unclear. Few benefits of mTICI-3 reperfusion were suggested by point estimates among patients with the highest onset-to-puncture time quartile, but the heterogeneity was not statistically significant. A recent study reported that the effect of complete reperfusion was retained among patients who underwent mechanical thrombectomy after 6 hours of stroke onset^26^. Onset-to-puncture time may be less critical than puncture-to-reperfusion time with respect to the effect of complete reperfusion, because onset-to-puncture time is sometimes imprecisely determined or documented^27^, while puncture-to-reperfusion time is generally accurately documented.

It is known that discrepancy exists between the assessment of the mTICI score by local operators and by independent core lab, although the degree of discrepancy varies^28,29^. As operators tend to overestimate the degree of reperfusion^28^, the successful (mTICI-2b/3) reperfusion rate in the RESCUE-Japan Registry 2 may be overestimated. In addition, more detailed grading scores including -2c (near-complete reperfusion) have been proposed to predict clinical outcomes precisely^30,31^. Those grading scores, which our cohort did not employ, may help to identify patients who can benefit from adding endovascular procedures. Further studies with core lab assessment and more detailed grading score are needed to clarify the limitation of the effect of complete reperfusion.

There are several limitations in this study. First, in this observational study, there might be unmeasured factors that inhibited complete reperfusion, and those could be confounded with the outcome. Second, assessments of mRS scores could be biased because mTICI scores were not blinded to physicians who rated mRS scores. Third, ischemic penumbra was not assessed. Finally, the heterogeneity in the effect of mTICI-3 reperfusion according to puncture-to-reperfusion time was not shown by the analysis where puncture-to-reperfusion time was included as a continuous variable. This may be because the relationship between clinical outcomes and puncture-to-reperfusion time is not linear, that is, the rate of favorable outcome decreases rapidly as time elapses in the early period of puncture-to-reperfusion time, and it reaches a plateau later^32^. The threshold of puncture-to-reperfusion time in this study is derived from our own registry, and its generalizability has not been confirmed. Therefore, it needs to be validated against other registries.

## Conclusions

mTICI-3 reperfusion, especially when achieved in less than 80 minutes, was superior to mTICI-2b reperfusion, regardless of baseline characteristics. Achieving early and complete reperfusion is important to improve outcomes. Until when operators should continue the procedure after mTICI-2b has been achieved, needs to be studied.

## Supplementary information

Supplementary Information.

## Data Availability

The datasets generated during and/or analyzed during the current study are not publicly available but are available from the corresponding author on reasonable request.
