# Supplementary material for: Futile complete recanalization: patients characteristics and its time course
Source: Sci Rep. 2020 Mar 18;10:4973. doi: 10.1038/s41598-020-61748-y (PMC7080727; doi:10.1038/s41598-020-61748-y)
Supplement: Supplementary file 1 — Supplementary Information. [file 41598_2020_61748_MOESM1_ESM.pdf]

Supplementary Information

Futile complete recanalization: patients characteristics and its time course

Takaya Kitano, Kenichi Todo, Shinichi Yoshimura, Kazutaka Uchida, Hiroshi Yamagami, Nobuyuki Sakai, Manabu Sakaguchi, Hajime Nakamura, Haruhiko Kishima, Hideki Mochizuki, Masayuki Ezura, Yasushi Okada, Kazuo Kitagawa, Kazumi Kimura, Makoto Sasaki, Norio Tanahashi, Kazunori Toyoda, Eisuke Furui, Yuji Matsumaru, Kazuo Minematsu, Takeshi Morimoto

Supplementary Methods

The name of institutional review boards of all participating centers are as follows: institutional review boards of Red Cross Ise Hospital, Ube Industries Central Hospital, Ogaki Tokushukai Hospital, Osaka Medical college, Osaka University Hospital, Kagawa University, Kawasaki Medical School Hospital, Kanazawa medical university hospital, Kitasato University, Gifu University, Kyushu Medical Center, Red Cross Kyoto Daiichi Hospital, Kinki University, Kurashiki Central Hospital, Kurume University Hospital, Kannan Hospital, Kobe City Medical Center General Hospital, Kokura Memorial Hospital, National Cerebral and Cardiovascular Center, Saiseikai Toyama Hospital, Saiseikai Nagasaki Hospital, Saitama Medical University International Medical Center, Sapporo Medical University, Shimizu Hospital, Juntendo University Hospital, Seisho Hospital, National Hospital Organization Sendai Medical Center, Koseikai Takeda Hospital, Tanushimaru Central Hospital, Tama Medical Center, Tokushima University, Toranomon Hospital, Nagoya University, Red Cross Nagoya Daini Hospital, Nippon Medical School, Hakodate Shintoshi Hospital, Hakodate Neurosurgical Hospital, Hyogo College of Medicine, Hyogo Brain and Heart Center, Hirosaki University, Hiroshima University, Red Cross Fukui Hospital, Fukuoka University Chikushi Hospital, Mazda Hospital, Mie University Hospital, Miyakonojo Medical Association Hospital, Yamaguchi Prefectural Grand Medical Center, Yamaguchi University and Yokohamashintoshi Neurosurgical Hospital.

Supplementary Tables

Supplementary Table S1. Patients baseline characteristics according to onset-to-reperfusion (O2R) time.

	O2R < 365 minutes		O2R ≥ 365 minutes	
	mTICI-3	mTICI-2b	mTICI-3	mTICI-2b
Age, years	74 (67–82)	75 (67–82)	79 (69–83)	79 (69–84)
Male gender	171 (56.4%)	143 (58.4%)	52 (60.5%)	55 (60.4%)
Smoking	44 (14.5%)	34 (13.9%)	11 (12.8%)	14 (15.4%)
Modified Rankin Scale score	0 (0–1)	0 (0–1)	0 (0–1)	0 (0–0)
Hypertension	174 (57.4%)	128 (52.2%)	51 (59.3%)	60 (65.9%)
Diabetes	60 (19.8%)	39 (15.9%)	14 (16.3%)	23 (25.3%)
Hyperlipidemia	68 (22.4%)	50 (20.4%)	19 (22.1%)	25 (27.5%)
Atrial fibrillation	172 (56.8%)	134 (54.7%)	42 (48.8%)	48 (52.7%)
NIHSS score	18 (14–23)	18 (14–23)	16 (13–21)	16 (11–22)
ASPECTS	8 (6–9)	7 (6–9)	8 (7–9)	7 (6–8)
Site of occlusion				
ICA	113 (37.3%)	80 (32.7%)	31 (36.0%)	33 (36.3%)
M1	139 (45.9%)	114 (46.5%)	48 (55.8%)	46 (50.5%)
M2 or distal	55 (18.2%)	48 (19.6%)	12 (14.0%)	15 (16.5%)
ACA	2 (0.7%)	9 (3.7%)	0 (0.0%)	1 (1.1%)
Cardioembolic stroke	249 (82.2%)	192 (78.4%)	60 (69.8%)	66 (72.5%)
Intravenous alteplase	196 (64.7%)	160 (65.3%)	7 (8.1%)	11 (12.1%)
Use of stent retrievers	208 (68.6%)	170 (69.4%)	59 (68.6%)	67 (73.6%)
Use of aspiration catheters	151 (49.8%)	141 (57.6%)	44 (51.2%)	51 (56.0%)
Onset to reperfusion, minutes	220 (160–275)	230 (175–280)	510 (430–660)	515 (435–615)
Onset to puncture, minutes	165 (115–225)	160 (115–205)	437.5 (365–620)	450 (360–545)
Puncture to reperfusion, minutes	40 (30–60)	55 (35–80)	60 (35–90)	55 (40–90)

Data are presented as n (%) or median (interquartile range). ACA, anterior cerebral artery; ASPECTS, Alberta Stroke Program Early Computerized Tomography Score; ICA, internal carotid artery; mTICI, modified treatment in cerebral infarction; NIHSS, National Institutes of Health Stroke Scale; M1, the horizontal segment of the middle cerebral artery; and M2, the insular segment of the middle cerebral artery.

Supplementary Table S2. Patients baseline characteristics according to onset-to-puncture (O2P) time.

	O2P < 305 minutes		O2P ≥ 305 minutes	
	mTICI-3	mTICI-2b	mTICI-3	mTICI-2b
Age, years	74 (67–82)	75 (67–82.5)	78 (68–83)	79 (70–84)
Male gender	173 (57.7%)	147 (59.3%)	50 (56.2%)	51 (58.0%)
Smoking	43 (14.3%)	34 (13.7%)	12 (13.5%)	14 (15.9%)
Modified Rankin Scale score	0 (0–1)	0 (0–1)	0 (0–1)	0 (0–0)
Hypertension	169 (56.3%)	131 (52.8%)	56 (62.9%)	57 (64.8%)
Diabetes	58 (19.3%)	40 (16.1%)	16 (18.0%)	22 (25.0%)
Hyperlipidemia	67 (22.3%)	50 (20.2%)	20 (22.5%)	25 (28.4%)
Atrial fibrillation	171 (57.0%)	135 (54.4%)	43 (48.3%)	47 (53.4%)
NIHSS score	18 (14–23)	18 (14–23)	16 (14–21)	17 (12.5–22)
ASPECTS	8 (6–9)	7 (6–9)	8 (7–9)	7 (6–8)
Site of occlusion				
ICA	111 (37.0%)	82 (33.1%)	33 (37.1%)	31 (35.2%)
M1	139 (46.3%)	115 (46.4%)	48 (53.9%)	45 (51.1%)
M2 or distal	54 (18.0%)	48 (19.4%)	13 (14.6%)	15 (17.0%)
ACA	2 (0.7%)	9 (3.6%)	0 (0.0%)	1 (1.1%)
Cardioembolic stroke	247 (82.3%)	194 (78.2%)	62 (69.7%)	64 (72.7%)
Intravenous alteplase	196 (65.3%)	163 (65.7%)	7 (7.9%)	8 (9.1%)
Use of stent retrievers	207 (69.0%)	173 (69.8%)	60 (67.4%)	64 (72.7%)
Use of aspiration catheters	151 (50.3%)	141 (56.9%)	44 (49.4%)	51 (58.0%)
Onset to reperfusion, minutes	220 (160–275)	230 (177.5–280)	505 (420–655)	522.5 (440–620)
Onset to puncture, minutes	160 (115–217.5)	162.5 (115–205)	430 (360–620)	450 (360–550)
Puncture to reperfusion, minutes	40 (30–60)	55 (35–80)	45 (30–70)	50 (37.5–90)

Data are presented as n (%) or median (interquartile range). ACA, anterior cerebral artery; ASPECTS, Alberta Stroke Program Early Computerized Tomography Score; ICA, internal carotid artery; mTICI, modified treatment in cerebral infarction; NIHSS, National Institutes of Health Stroke Scale; M1, the horizontal segment of the middle cerebral artery; and M2, the insular segment of the middle cerebral artery.

Supplementary Table S3. Patients baseline characteristics according to puncture-to-reperfusion (P2R) time.

	P2R < 80 minutes		P2R ≥ 80 minutes	
	mTICI-3	mTICI-2b	mTICI-3	mTICI-2b
Age, years	75 (67–82)	76 (68–82)	79 (69–85)	75 (65–84)
Male gender	190 (58.5%)	135 (57.0%)	33 (51.6%)	63 (63.6%)
Smoking	47 (14.5%)	33 (13.9%)	8 (12.5%)	15 (15.2%)
Modified Rankin Scale score	0 (0–0)	0 (0–0)	0 (0–1)	0 (0–1)
Hypertension	189 (58.2%)	125 (52.7%)	36 (56.3%)	63 (63.6%)
Diabetes	63 (19.4%)	41 (17.3%)	11 (17.2%)	21 (21.2%)
Hyperlipidemia	69 (21.2%)	51 (21.5%)	18 (28.1%)	24 (24.2%)
Atrial fibrillation	182 (56.0%)	132 (55.7%)	32 (50.0%)	50 (50.5%)
NIHSS score	17 (14–22)	18 (14–22)	18.5 (14–23)	18 (13–23)
ASPECTS	8 (6–9)	7 (6–8)	7 (6–9)	8 (6–9)
Site of occlusion				
ICA	113 (34.8%)	74 (31.2%)	31 (48.4%)	39 (39.4%)
M1	163 (50.2%)	111 (46.8%)	24 (37.5%)	49 (49.5%)
M2 or distal	54 (16.6%)	51 (21.5%)	13 (20.3%)	12 (12.1%)
ACA	2 (0.6%)	7 (3.0%)	0 (0.0%)	3 (3.0%)
Cardioembolic stroke	261 (80.3%)	194 (81.9%)	48 (75.0%)	64 (64.6%)
Intravenous alteplase	179 (55.1%)	127 (53.6%)	24 (37.5%)	44 (44.4%)
Use of stent retrievers	229 (70.5%)	162 (68.4%)	38 (59.4%)	75 (75.8%)
Use of aspiration catheters	145 (44.6%)	128 (54.0%)	50 (78.1%)	64 (64.6%)
Onset to reperfusion, minutes	230 (165–320)	235 (175–350)	335 (265–427.5)	320 (250–435)
Onset to puncture, minutes	190 (125–285)	185 (130–310)	212.5 (142.5–312.5)	195 (140–335)
Puncture to reperfusion, minutes	35 (30–50)	45 (35–55)	110 (90–135)	105 (90–140)

Data are presented as n (%) or median (interquartile range). ACA, anterior cerebral artery; ASPECTS, Alberta Stroke Program Early Computerized Tomography Score; ICA, internal carotid artery; mTICI, modified treatment in cerebral infarction; NIHSS, National Institutes of Health Stroke Scale; M1, the horizontal segment of the middle cerebral artery; and M2, the insular segment of the middle cerebral artery.

Supplementary Figures

Subgroups		No. of patients mTICI-3	No. of patients mTICI-2b	OR	95% CI	P for interaction
Age	≤ 75	197	160	2.31	1.41–3.78	0.38
	> 75	192	176	2.19	1.19–4.05	
Alteplase	Yes	203	171	2.29	1.39–3.78	0.62
	No	186	165	1.97	1.09–3.55	
NIHSS	≥ 10	351	297	2.21	1.47–3.32	0.54
	< 10	38	39	2.83	0.99–8.14	
ASPECTS	≥ 6	332	264	2.29	1.55–3.39	0.72
	< 6	57	72	2.20	0.64–7.61	
ICA or M1		332	268	2.24	1.45–3.45	0.35
M2 or distal*		67	68	1.57	1.02–4.74	

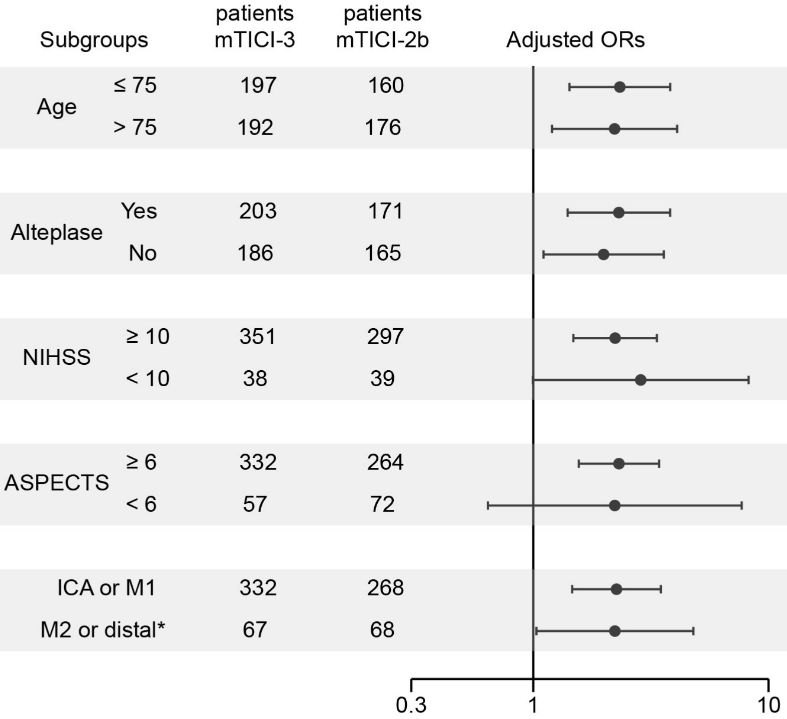

Supplementary Fig. S1. Adjusted odds ratios (ORs) of complete reperfusion (mTICI-3) for excellent outcome according to baseline characteristics. Adjusted for age, sex, NIHSS score, ASPECTS, target occlusion location, intravenous alteplase administration, and onset-to-reperfusion time.

* Five patients with anterior cerebral artery occlusion are included.

NIHSS, National Institutes of Health Stroke Scale; ASPECTS, Alberta Stroke Program Early Computerized Tomography Score; CI, confidence interval; ICA, internal carotid artery; mTICI, modified thrombolysis in cerebral infarction; M1, the horizontal segment of the middle cerebral artery; M2, the insular segment of the middle cerebral artery.

Subgroups		No. of patients mTICI-3	No. of patients mTICI-2b	Adjusted ORs	OR	95% CI	P for interaction
Onset to reperfusion	< 365	303	245		2.35	1.55–3.55	0.68
	≥ 365	86	91		1.66	0.69–4.03	
Onse to Puncture	< 305	300	248		2.13	1.39–3.26	0.87
	≥ 305	89	88		1.40	0.57–3.48	
Puncture to reperfusion	< 80	325	237		2.35	1.53–3.61	0.073
	≥ 80	64	99		1.17	0.46–3.00	

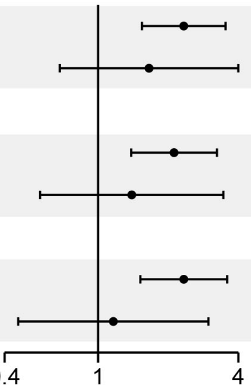

Supplementary Fig. S2. Adjusted odds ratios (ORs) of complete reperfusion (mTICI-3) for excellent outcome according to time course (onset-to-reperfusion, onset-to-puncture, and puncture-to-reperfusion time). These time variables are divided by the upper quartile.

CI; confidence interval; mTICI, modified thrombolysis in cerebral infarction.

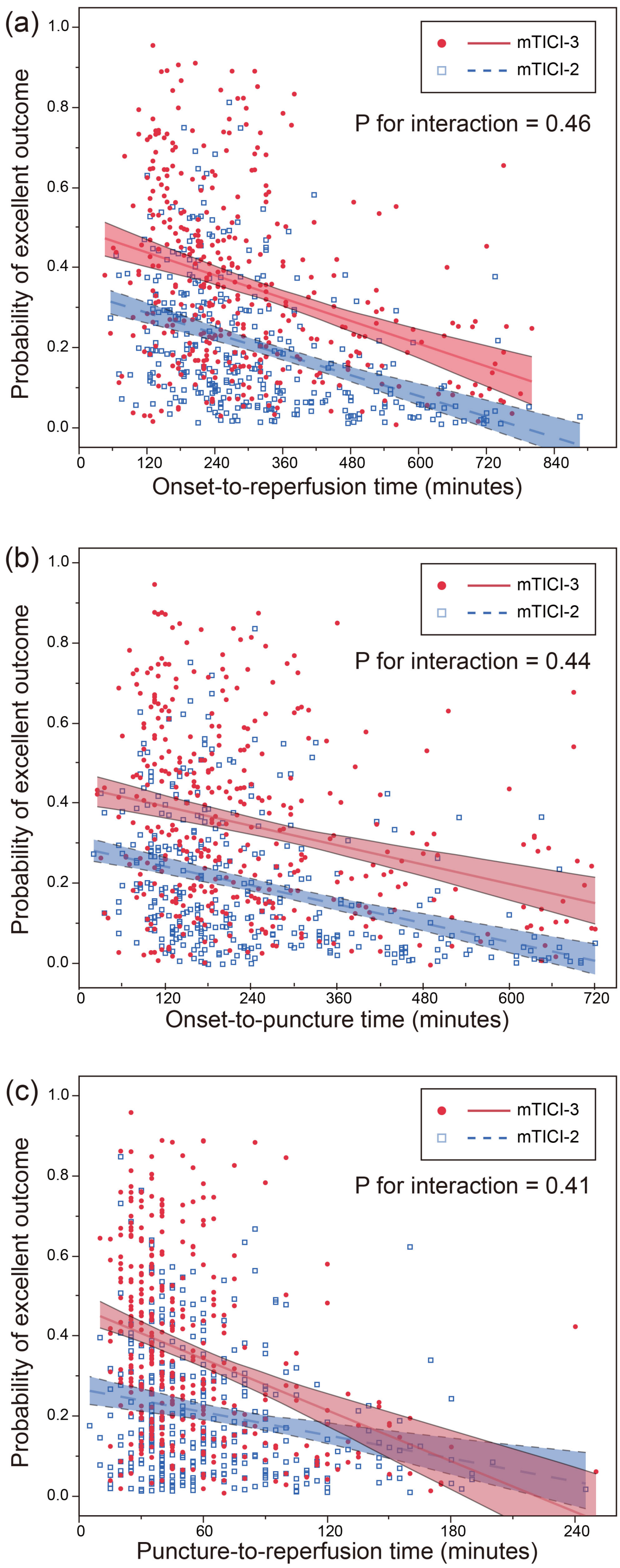

Supplementary Fig. S3. The probabilities of excellent outcome estimated using logistic regression with stratification by mTICI scores are plotted. The regression lines and 95% confidence intervals are shown. The time course was included as a continuous variable. As a continuous variable, none of the onset-to-reperfusion time, onset-to-puncture time, or puncture-to-reperfusion time significantly modified the effect of complete reperfusion. (a) On onset-to-reperfusion time. Adjusted for baseline characteristics (age, sex, National Institutes of Health Stroke Scale score, Alberta Stroke Program Early Computerized Tomography Score, target occlusion location, intravenous alteplase). (b) On onset-to-puncture time. Adjusted for baseline characteristics and puncture-to-reperfusion time. (c) On puncture-to-reperfusion time. Adjusted for baseline characteristics and onset-to-puncture time.
